# Supplementary material for: Synergistic effect of two human-like monoclonal antibodies confers protection against orthopoxvirus infection
Source: Nat Commun. 2024 Apr 16;15:3265. doi: 10.1038/s41467-024-47328-y (PMC11021552; doi:10.1038/s41467-024-47328-y)
Supplement: Supplementary file 1 — Supplementary Information [file 41467_2024_47328_MOESM1_ESM.pdf]

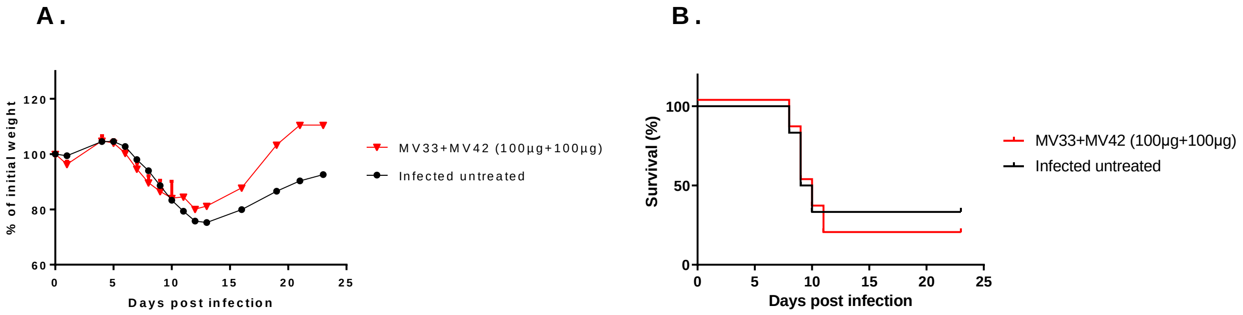

Supplementary Figure 1. Therapeutic efficacy of single dose mAb treatment against lethal ECTV infection at 6 days post infection. Body weight changes (A) and survival rates (B) of BALB/c mice infected with ECTV (50 PFU i.n.) and treated with MV33+EV42 (100+100µg) or left untreated (infected untreated). n= 6 / group. For (A) measurement data are expressed as mean + standard error (SE).

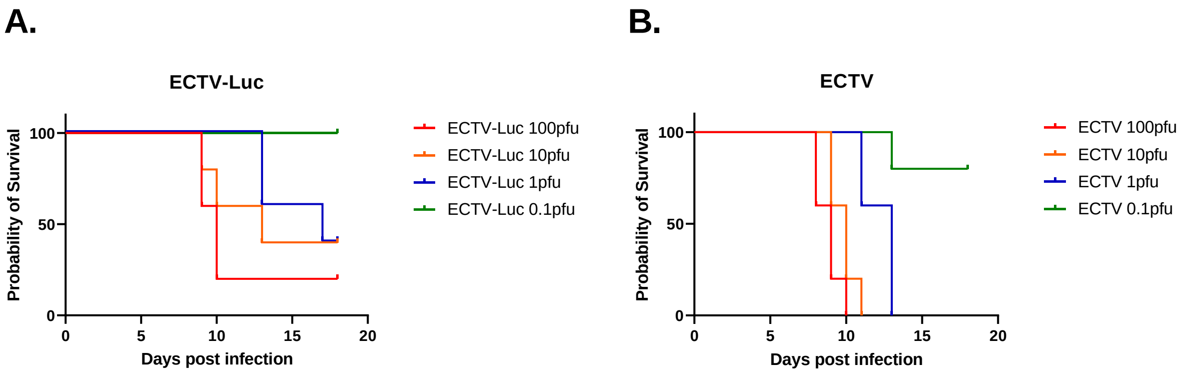

LD_{50} calculated ratio: 4.02 pfu (ECTV-Luc) / 0.58 pfu (ECTV) = 7

Supplementary Figure 2. Lethal dose evaluation of ECTV-Luc and ECTV. Survival rates of BALB/c mice were infected with either ECTV-Luc (A) or ECTV (B) at the indicated doses. The LD50 values for each strain and the ratio between these values were calculated. n= 5 / group.
